# Altered Language-Related Effective Connectivity in Patients with Benign Childhood Epilepsy with Centrotemporal Spikes

**DOI:** 10.3390/life13020590

**Published:** 2023-02-20

**Authors:** Fei Yang, Juan Tan, Yue Huang, Ruhui Xiao, Xiaoming Wang, Yanbing Han

**Affiliations:** 1 Department of Neurology, First Affiliated Hospital of Kunming Medical University, Kunming 650051, China; 2 Department of Neurology, Affiliated Hospital of North Sichuan Medical College, Nanchong 637503, China; 3 Department of Pediatrics, Affiliated Hospital of North Sichuan Medical College, Nanchong 637503, China; 4 Department of Radiology, Affiliated Hospital of North Sichuan Medical College, Nanchong 637503, China

**Keywords:** benign epilepsy with centrotemporal spikes, effective connectivity, Granger causality analysis, Broca’s area, language network

## Abstract

Benign childhood epilepsy with centrotemporal spikes (BECTS) is one of the most common childhood epilepsy syndromes and may be associated with language deficits. Resting-state functional magnetic resonance imaging (fMRI) data were collected from a total of 78 children: 52 patients with BECTS (28 drug-naïve and 24 medicated) and 26 healthy controls (HC). Granger causality analysis (GCA) was used to investigate alterations in effective connectivity (EC) between the language network core node (Broca’s area) and the whole brain. EC from Broca’s area to the left Heschl’s gyrus (HG), right putamen, and anterior cingulate cortex (ACC) was significantly increased, while EC from the bilateral putamen and left ACC to Broca’s area was significantly decreased in BECTS. Moreover, altered EC of Broca’s area to the right putamen was significantly positively correlated with verbal IQ (VIQ), while altered EC of Broca’s area to the ACC showed significantly negative correlations with the frequency of seizures. Altered EC from the left putamen to Broca’s area was also significantly negatively correlated with performance IQ (PIQ) and full-scale IQ (FSIQ) in the drug-naïve group. In addition, there was a significant positive correlation between the EC of Broca’s area to the left HG and the number of seizures, as well as between the EC of Broca’s area to the right putamen and the age at onset in the medicated group. These findings suggest abnormal causal effects on the language network related to Broca’s area in children with BECTS. Longitudinal investigation of language network development and further follow-up may be needed to illuminate the changes in organization and rebalancing over time.

## 1. Introduction

Benign epilepsy of childhood with centrotemporal spikes (BECTS) is one of the most common types of focal epilepsy among children [1]. The onset age ranges from 3 to 13 years, with a peak at 9 to 10 years old [2]. Although clinical episodes are infrequent, approximately 90% of BECTS and ABPE (atypical benign partial epilepsy of childhood, which can be evolved by BECTS) patients have atypical electrical status epilepticus in sleep (ESES) and almost 75% show spikes in the Rolandic region [3]. With advances in neuropsychology research, the “benign” concept in BECTS has been challenged because some patients may suffer from some degree of cognitive impairment, especially language dysfunction [4,5]. Recent cross-sectional studies found that 54% of BECTS children showed language deficits and 42% had dyslexia [6]. Monjauze et at. [7] also found that BECTS children in remission had persistent deficits in linguistic skills, suggesting possible long-term effects during the course of epilepsy. Cognitive dysfunction may be related to abnormal brain function changes caused by epileptic activity. Therefore, it is very important to explore relevant cognitive impairment mechanisms in BECTS to protect patients’ cognitive function.

Functional magnetic resonance imaging (fMRI) has become an increasingly important method of studying brain function because of its noninvasive nature and high spatial resolution for studying brain networks [8,9,10]. Resting-state fMRI allows us to identify significant baseline fluctuations to obtain task-free functional network information and to identify epileptic circuits, providing clinicians with clues about the core functional regions of epileptic activity [11]. In this resting state, the brain regions with low-frequency fluctuations are called the resting-state network (RSN), such as the visual network, auditory network and default mode network (DMN). The RSN may represent underlying or intrinsic functional connectivity in the brain [12]. Studies on BECTS in recent years have investigated many RSNs, such as the sensorimotor network (SMN) [13], DMN [14] and attention network [15]; of those, the language network is of particular interest because of language problems associated with BECTS [6,16,17]. The functional connectivity between the language network and the SMN [18], motor network [19] or DMN [20] in BECTS patients is decreased. However, these linear correlations do not provide information about the direction of causality between regions [21]. Further research is also needed to determine which functional brain regions exert causal effects on language areas.

Effective connectivity (EC) provides more structural information related to synaptic density and strength and is also determined by the excitability of each brain region, revealing directional (causal) interactions between brain regions [22]. Granger causality analysis (GCA) is a directed functional connectivity method based on coefficients used to identify causal connectivity in neural time-series data. GCA quantifies the magnitude and direction of the influence of one regional time series on another regional time series [23]. Brain regions contributing to changes in the region of interest (ROI) and the size of the effect can be identified, which is conducive to further understanding seizure-induced damage [24]. Functionally, Broca’s area is responsible for speech production. Activation patterns in Broca’s area have also been associated with various types of language tasks in fMRI studies [25]. In the current study, we identified brain regions with altered EC with the language network in BECTS patients, aiming to enhance current clinical understanding. We hypothesized that the ECs between the language network core node (Broca’s area) and the peripheral language areas are changed in BECTS, and that the altered EC is related to the clinical characteristics. Therefore, GCA was used to analyze the EC between different brain regions and language networks by setting Broca’s area as the ROI.

## 2. Materials and Methods

### 2.1. Subjects

A total of 78 right-handed children (aged 6–16 years) were enrolled in the study. Fifty-two patients with BECTS were recruited between June 2012 and April 2016 from the Pediatric Clinic of the Affiliated Hospital of North Sichuan Medical College: 28 drug-naïve and 24 medicated BECTS patients. In standard evaluations, a medical history, physical and neurological examination, long-term electroencephalogram (EEG) and brain MRI scan were conducted to determine the diagnosis and differential diagnosis. International League Against Epilepsy criteria [26,27] were used to diagnose BECTS. The inclusion criteria were as follows: (a) seizures mainly manifested on one side of the face or as brief mouth motor seizures, often accompanied by somatosensory symptoms, often with nighttime onset, and tended to generalize; (b) blunt centrotemporal spikes appeared on the EEG and were often followed by slow waves that tended to spread or shift; (c) patients were aged 6–16 years; and (d) patients were right-handed. We defined the time from the first definite seizure to MRI acquisition as the epilepsy disease duration. If blunt centrotemporal spikes appeared only on the left cerebral side on the EEG, BECTS was considered left (L)-lateralized; if they appeared only on the right cerebral side, BECTS was considered right (R)-lateralized; if they appeared on both sides, BECTS was considered bilateral (B). The exclusion criteria were as follows: (a) patients with other neurological psychological disorders that may impair cognitive function; (b) patients with traumatic brain injury; (c) patients who were unable to undergo an MRI scan for other reasons, such as claustrophobia or metal implants; and (d) detection of structural cerebral lesions on MRI. Twenty-six age- and sex-matched subjects from school were also recruited in the present study as healthy controls (HCs).

### 2.2. Psychometric Tests

Intelligence quotient (IQ) scores, including verbal IQ (VIQ), performance IQ (PIQ) and full-scale IQ (FSIQ), were obtained for all subjects with the Chinese version of the Wechsler Intelligence Scale for Children (WISC-III).

### 2.3. Image Data Acquisition

MRI scans were collected by a 3-Tesla GE Discovery MR scanner with an 8-channel head coil at the Affiliated Hospital of North Sichuan Medical College. T1-weighted images were acquired using a three-dimensional fast spoiled gradient-echo sequence. The parameters were repetition time (TR) = 6.008 ms, echo time (TE) = 1.984 ms, matrix size = 256 × 256, flip angle (FA) = 9°, field of view (FOV) = 256 mm × 256 mm, voxel size = 0.94 mm × 0.94 mm × 1 mm, and slice thickness (no gap) = 1 mm. During the whole resting-state scan, the subjects were instructed to close their eyes, relax and avoid thinking about anything in particular, and stay awake. Resting-state functional data were acquired using an echo-planar imaging sequence. The parameters were as follows: TE = 30 ms, FOV = 240 mm × 240 mm; voxel size = 3.75 mm × 3.75 mm × 4 mm, TR = 2000 ms, FA = 90°, matrix size = 64 × 64, slice thickness = 4 mm, slice spacing = 0.4 mm, number of slices = 35, slice order = interleaved, and view order = bottom-up. The scan lasted for 410 s for each participant, and 205 volumes were generated.

### 2.4. MRI Date Preprocessing

The software package NIT (http://www.neuro.uestc.edu.cn/NIT.html (accessed on 5 February 2022)) was used to preprocess the resting-state fMRI data. The steps included the following: (a) removal of the first five time points; (b) slice-timing correction; (c) realignment and exclusion of participants with maximum motion exceeding 3 mm or/and 3°; (d) spatial normalization to the Montreal Neurological Institute(MRI) template and resampling of the voxel size into 3 mm × 3 mm × 3 mm; (e) spatial smoothing (with a full width at half maximum (FWHM) = 8 mm); (f) regressed out white-matter signals, cerebrospinal fluid signals, and 12 head-motion parameters; (g) filtered (0.01–0.08 Hz); (h) the linear drifts were removed. Eight BECTS patients and 3 HC subjects were excluded due to head motion exceeding 3 mm and/or 3°. The final analysis included 22 drug-naïve patients (11 male/11 female, age: 9.11 ± 1.86 years), 22 medicated patients (16 male/6 female, age: 9.68 ± 1.73 years), and 23 HC (16 male/7 female, age: 10.09 ± 2.86 years).

### 2.5. Granger Causality Analysis

A spherical region (radius of 5 mm) located in the left Broca’s area (Brodmann area 44 (−53, 12, 19)) was selected as the ROI. Then, the REST (http://www.restfmri.net/forum/REST (accessed on 16 February 2022)) software package was used to conduct GCA. The coefficient-based GCA was performed, and its regressive model was as follows:

$$Y_{t}=\sum_{k=1}^{k=P}A_{k}X_{\left(t-k\right)}+\sum_{k=1}^{k=P}B_{k}Y_{\left(t-k\right)}+CZ_{t}+E_{t}$$

$$X_{t}=\sum_{k=1}^{k=P}A_{k}^{’}X_{\left(t-k\right)}+\sum_{k=1}^{k=P}B_{k}^{’}Y_{\left(t-k\right)}+C^{\prime }Z_{t}+E_{t}^{’}$$

where the time series $X_{t}$ was the average time series of the ROI, and the time series $Y_{t}$ was the time series in each voxel of the whole brain. We calculated the voxel-wise GCA from $X_{t}$ to $Y_{t}$, and from $Y_{t}$ to $X_{t}$. $A_{k}$, $A_{k}^{’}$, $B_{k}$ and $B_{k}^{’}$ were the regression coefficients. C and $C^{\prime }$ were the covariable coefficients. $E_{t}$ and $E_{t}^{’}$ were serially uncorrelated residuals. *P* was the model order, which was set as 1 here. $Z_{t}$ was the covariable. Thus, the coefficient $A_{k}$ represented the causality of ROI(X) to voxel(Y), and $A_{k}^{’}$ represented the causality of voxel (Y) to ROI (X). Finally, the z score transformation was applied to the GCA maps ($A_{k}$ and $A_{k}^{’}$).

### 2.6. Statistical Analysis

The one-sample *t* test in SPM 12 was used to assess the within-group functional connectivity density (FCD) maps in each group (*p* < 0.05, false discovery-corrected). One-way ANOVA (*p* < 0.05, AlphaSim corrected) with post hoc analysis (Sidak test) was performed to compare the GCA differences among groups. Partial correlation analysis was used to analyze the correlations between the altered GCA and the clinical information, controlling for both age and sex.

## 3. Results

### 3.1. Demographic Characteristics and Neuropsychological Tests

There were no significant differences in age (*p* = 0.343) or sex (*p* = 0.233) among the three groups. There was no significant difference in age at onset (*p* = 0.350), EEG lateralization (*p* = 0.324), or frequency of seizures (*p* = 0.381) between the medicated group and the drug-naïve group. There were significant differences in the duration of disease (*p* < 0.05) and the number of seizures (*p* < 0.05) between the medicated group and the drug-naïve group. The VIQ, PIQ and FSIQ scores of BECTS patients (both drug-naïve and medicated) were significantly lower than those of the HC group (*p* < 0.001). No significant difference was found between the drug-naïve group and the medicated group. Demographic characteristics and IQ scores are shown in Table 1.

### 3.2. Differences in GCA among Drug-Naïve, Medicated, and HCs

**Figure 1:**
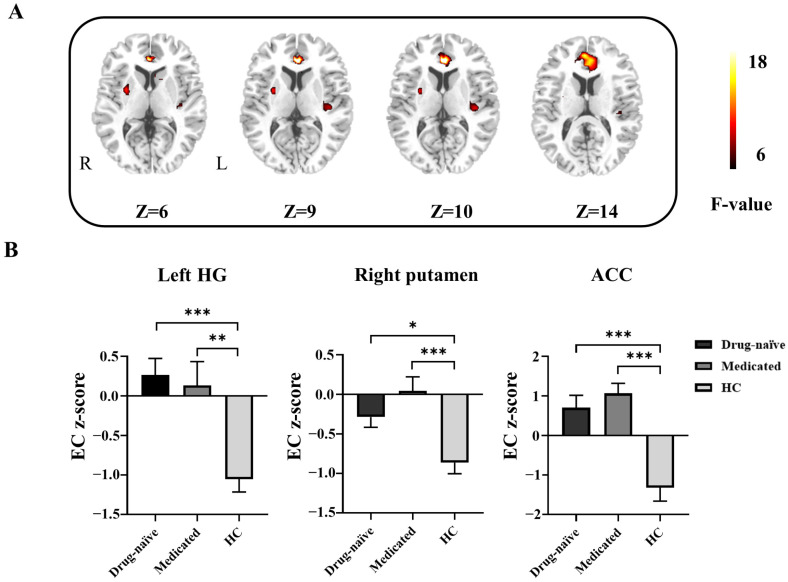
Differences in effective connectivity (EC) from Broca’s area to every whole-brain voxel among the groups. (**A**) Brain regions with significant group differences according to one-way ANOVA and post hoc analyses. R, right; L, left. (**B**) Bar graph showing the ROI-wise post hoc analysis results. * *p* < 0.05; ** *p* < 0.01; *** *p* < 0.001. HG, Heschl’s gyrus; ACC, anterior cingulate cortex; drug-naïve, unmedicated benign epilepsy with centrotemporal spikes; medicated, medicated benign epilepsy with centrotemporal spikes; HC, healthy control group.

As shown in Table 2, there were significant differences in the left Heschl’s gyrus (HG), right putamen and anterior cingulate cortex (ACC) among the three groups according to the ANOVA results. In the post hoc analyses, the BECTS patients (both drug-naïve and medicated) showed increased EC from Broca’s area to the left HG, right putamen, and ACC compared with the HC group. No significant difference was found between the drug-naïve group and the medicated group (Figure 1).

**Figure 2:**
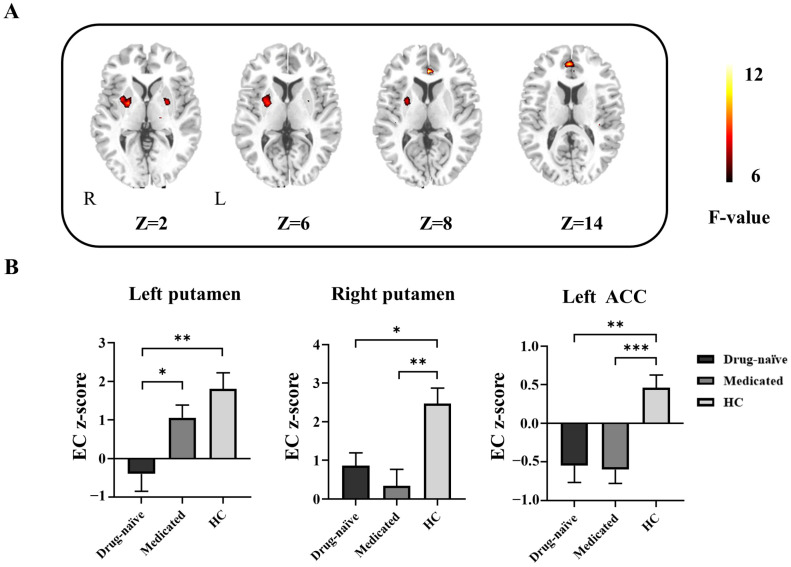
Differences in effective connectivity (EC) from every whole-brain voxel to Broca’s area among groups. (**A**) Brain regions with significant group differences according to one-way ANOVA and post hoc analyses. R, right; L, left. (**B**) Bar graph showing the ROI-wise post hoc analysis results. * *p* < 0.05; ** *p* < 0.01; *** *p* < 0.001. ACC, anterior cingulate cortex; drug-naïve, unmedicated benign epilepsy with centrotemporal spikes; medicated, medicated benign epilepsy with centrotemporal spikes; HC, healthy control group.

According to the ANOVA, there was a significant main effect of group on the EC from the left putamen, right putamen and left ACC to Broca’s area (Table 3). Compared with the HC group, the BECTS patients (both drug-naïve and medicated) exhibited a decrease in the EC from the right putamen and left ACC to Broca’s area according to post hoc analysis. Decreased EC from the left putamen to Broca’s area was found in the drug-naïve group compared with the medicated group (Figure 2).

### 3.3. Abnormal ECs Was Correlated with Some Clinical Features

**Figure 3:**
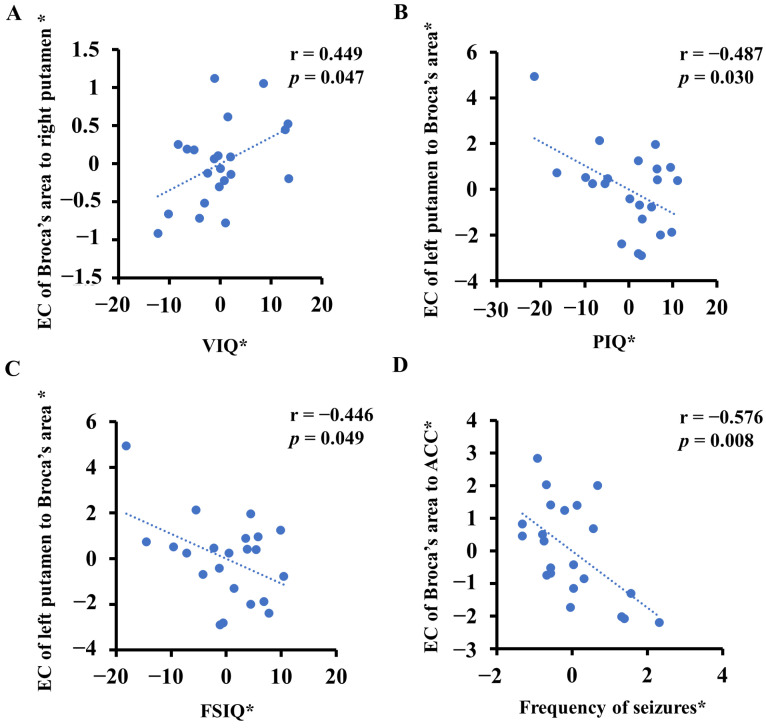
Correlations between clinical features and abnormal effective connectivity (EC) in the drug-naïve group (unmedicated benign epilepsy with centrotemporal spikes). Asterisks (*) indicate residuals of the linear regression model controlling for both age and sex. (**A**) The EC of Broca’s area to the right putamen showed significantly positive correlations with VIQ (verbal intelligence quotient). (**B**) The EC from the left putamen to Broca’s area showed significantly negative correlations with PIQ (performance intelligence quotient). (**C**) The EC from the left putamen to Broca’s area showed significantly negative correlations with FSIQ (full-scale intelligence quotient). (**D**) The EC of Broca’s area to the ACC showed significantly negative correlations with the frequency of seizures. ACC, anterior cingulate cortex.

In the drug-naïve group, after controlling for the influence of gender and age, the EC of Broca’s area to the right putamen showed significantly positive correlations with VIQ (r = 0.449, *p* = 0.047) (Figure 3A). The EC from the left putamen to Broca’s area showed significantly negative correlations with the PIQ (r = −0.487, *p* = 0.030) (Figure 3B) and FSIQ (r = −0.446, *p* = 0.049) (Figure 3C). The EC of Broca’s area to the ACC showed significantly negative correlations with the frequency of seizures (r = −0.576, *p* = 0.008) (Figure 3D). The other variables (age, age at onset, disease duration and number of seizures) were not significantly correlated with abnormal EC values at *p* > 0.05.

**Figure 4:**
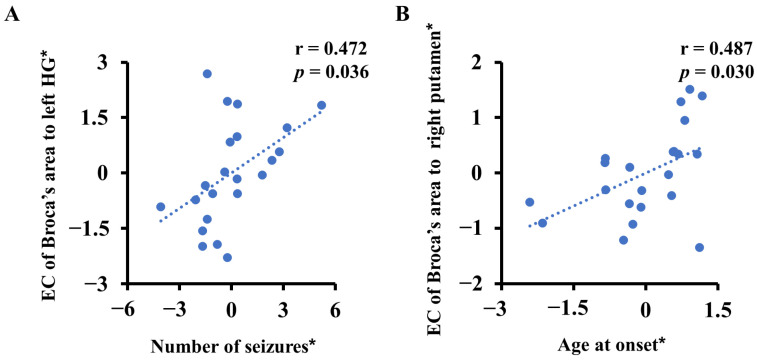
Correlations between clinical features and abnormal effective connectivity (EC) in the medicated group (medicated benign epilepsy with centrotemporal spikes). Asterisks (*) indicate residuals of the linear regression model controlling for both age and sex. (**A**) The EC from Broca’s area to the left HG was positively related to the frequency of seizures. HG, Heschl’s gyrus. (**B**) The EC from Broca’s area to the right putamen was positively related to the age at onset.

In the medicated group, the EC from Broca’s area to the left HG was positively related to the number of seizures (r = 0.472, *p* = 0.036) (Figure 4A). The EC from Broca’s area to the right putamen was significantly positively correlated with the age at onset (r = 0.487, *p* = 0.030) (Figure 4B). The other variables (VIQ, PIQ, age, disease duration and frequency of seizures) were not significantly correlated with abnormal EC values at *p* > 0.05.

## 4. Discussion

In the current study, changes in the EC between the language network core node (Broca’s area) and the whole brain in BECTS patients were determined by GCA. There were three major findings: (1) the EC from Broca’s area to the left HG, right putamen, and ACC were significantly increased, EC from bilateral putamen and left ACC was significantly increased, and EC from bilateral putamen and left ACC to Broca’s area was decreased in BECTS patients; (2) altered EC of Broca’s area to the right putamen was significantly positively correlated with VIQ, altered EC of Broca’s area to ACC was significantly negatively correlated with the frequency of seizures, and altered EC from left putamen to Broca’s area was significantly negatively correlated with PIQ and FSIQ in the drug-naïve group; and (3) there were positive correlations between the EC of Broca’s area to left HG and the number of seizures as well as between the EC of Broca’s area to the right putamen and age at onset in the medicated group.

GCA has been widely used to analyze EEG and fMRI signals in patients with epilepsy, which helps us to better understand seizure activity initiation, propagation, and termination and illuminates the data flow between the epilepsy networks [28,29]. A recent theoretical framework proposed that functional interactions between brain regions may change over time, and the core–peripheral model of language proficiency briefly involved in language processing may help with the processing of language tasks [30,31,32]. In this study, BECTS patients had abnormal EC between the language network core node (Broca’s area) and whole-brain voxels mainly in peripheral regions (the left HG and ACC) and subcortical regions (the bilateral putamen), similar to the results of a previous study in newly diagnosed BECTS patients [29]. Although the brain regions with altered EC were also cortical and subcortical, the specific brain regions differed, which may be due to inconsistency in the selected ROI [29]. Long-term epileptiform discharges are able to interrupt the brain’s normal function in BECTS patients [33] and can also cause disrupted EC between the brain regions associated with language and the language core node, which may contribute to the language impairment.

The left frontotemporal area, particularly Broca’s area and the left middle temporal gyrus (MTG), plays an integral role in language circuits [34]. In this study, the EC from Broca’s area to the left HG was increased in BECTS patients (both drug-naïve and medicated). It is well known that the centrotemporal spikes of BECTS persist longer than the seizures and are able to disrupt brain activity in the temporal gyrus [35,36]. Moreover, the EC of Broca’s area to the left HG was partially improved in the medicated group. There was also a significant positive correlation between abnormal EC and the number of seizures in the medicated group, which indicated that abnormal EC could be improved by effective antiseizure medications (ASMs) and reductions in the number of seizures. A previous study of fMRI and verbal working memory tasks in BECTS patients also found that the greater the number of seizures, the greater the abnormal activation of these regions [37]. It was also found that the association between altered EC strength and cognitive impairment (of verbal memory and language function) was affected by both seizure frequency and lateralization in patients with temporal lobe epilepsy (TLE) [21]. Our study also found that children with BECTS had lower VIQ, PIQ and FSIQ scores, which was consistent with the results of a previous study [5]. These findings suggest an interaction effect between epileptic area and seizures on neuroimaging features and cognitive function.

Our study also found increased EC from Broca’s area to the ACC and decreased EC from the left ACC back to Broca’s area in BECTS. Moreover, a significant negative correlation was found between the EC from Broca’s area to the ACC and the frequency of seizures in the drug-naïve group. The ACC is implicated in cognitive processes, including the mediation of executive function and the monitoring of conflict [38], and coordinates switching between the DMN and CEN (central executive network) depending on the individual’s needs [39]. Numerous studies have found that cognitive control during language processing is linked to frontal lobe regions, including the ACC and left ventrolateral prefrontal cortex (VLPFC) [40,41]. Therefore, abnormal changes in the EC between the ACC and Broca’s region may alter the regulation of language production and affect language function.

Under the current perspective, the putamen plays an important role in motor processing and executive function, because it receives projections from the motor and motor association cortices [42]. In this study, the EC from Broca’s area to the right putamen was significantly increased and the EC from the right putamen to Broca’s area was significantly decreased in BECTS patients, while the EC from the left putamen to Broca’s area was significantly decreased in the drug-naïve group compared with the medicated group and the HC group. In a previous study, putamen hyperplasia was found in BECTS patients, and putamen volume was significantly negatively correlated with age at onset, suggesting that abnormal cognitive control and executive function are related to developmental abnormalities [42]. Regarding Broca’s area and the putamen, our GCA indicated that frontostriatal connectivity differs in BECTS patients, which suggests disruption of the frontostriatal network. According to recent research, the frontostriatal circuit is involved in processing emotions, focusing and filtering cortical input, and selecting between cognitive and behavioral representations [43]. Moreover, the EC of Broca’s area to the right putamen was significantly positively correlated with VIQ in the drug-naïve group and with age at onset in the medicated group. The EC of the left putamen to Broca’s area was significantly negatively correlated with PIQ and FSIQ scores in the drug-naïve group. It is suggested that abnormal ECs are correlated with the onset of epileptic activity and cognitive function, but these abnormal ECs may be ameliorated by inhibitory factors during early development. Basal ganglia gamma-aminobutyric acid (GABA)ergic neurons have a direct inhibitory effect on epileptic discharge [44]. Abnormal striatal development caused by early onset of the disease may increase the inhibitory effect of GABAergic neurons, thus improving the abnormal EC of the frontostriatal circuit. Therefore, the EC of the abnormal frontostriatal circuit may be helpful in understanding cognitive abnormalities, especially language abnormalities, in BECTS patients.

The limitations of this study should be mentioned. First, it did not examine the longitudinal development of the language network, and further longitudinal follow-up may be needed to elucidate the changes in composition reorganization and rebalancing over time. Second, we did not observe clinical seizures in all patients during fMRI scans. However, since the EEG was not performed simultaneously with the fMRI scan, interictal epileptic discharges may have affected the fMRI data.

## 5. Conclusions

In summary, we found that BECTS patients had abnormal EC between the language network core node (Broca’s area) and peripheral language regions (the left HG and ACC) or subcortical regions (the bilateral putamen). Our study demonstrated that the language system imbalance in patients with BECTS and the possible rebalancing of the language system under these conditions might be through abnormal EC. Abnormal EC with Broca’s area might cause impaired language function in BECTS patients. Moreover, children with BECTS presented lower VIQ, PIQ and FSIQ scores, which might be associated with the disease’s epileptic activity. Abnormal EC was also correlated with some clinical features. These results suggest that epileptic activity might be involved in these abnormal driving effects. Subsequent longitudinal studies may better illuminate the course of BECTS, which will contribute to the understanding of brain development under pathological conditions.

## Figures and Tables

**Table 1:** Demographic characteristics and clinical features of the BECTS patients and healthy controls (mean ± SD).

Clinical Features	Drug-Naïve (*n* = 22)	Medicated (*n* = 22)	HCs (*n* = 23)	*p* Value
Age (year)	9.11 ± 1.86	9.68 ± 1.73	10.09 ± 2.86	0.343 ^a^
Sex (male/female)	11:11	16:6	16:7	0.233 ^b^
Age at onset (year)	8.64 ± 1.82	8.10 ± 2.06	-	0.350 ^c^
Duration (month)	6.58 ± 7.61	20.82 ± 11.82	-	<0.05 ^c^
EEG lateralization	9 ^L^/3 ^R^/10 ^B^	6 ^L^/7 ^R^/9 ^B^	-	0.324 ^b^
Number of seizures (count)	2.86 ± 1.35	4.14 ± 2.10	-	<0.05 ^c^
Frequency of seizures (counts/year)	2.55 ± 1.01	2.89 ± 1.50	-	0.381 ^c^
VIQ	84.00 ±7.35	82.41 ± 7.43	98.17 ± 6.04	<0.001 ^a^
PIQ	95.00 ± 10.71	93.18 ± 12.12	110.87 ±7.23	<0.001 ^a^
FSIQ	88.09 ± 8.66	86.13 ± 9.41	105.39 ± 6.04	<0.001 ^a^

Abbreviations: BECTS, benign childhood epilepsy with centrotemporal spikes; SD, standard deviation; drug-naïve, unmedicated benign epilepsy with centrotemporal spikes; medicated, medicated benign epilepsy with centrotemporal spikes; HCs, healthy controls; EEG, electroencephalogram; L, left; R, right; B, bilateral; VIQ, verbal intelligence quotient; PIQ, performance intelligence quotient; FSIQ, full-scale intelligence quotient; ANOVA, analysis of variance. Superscripts indicate statistical test: ^a^ ANOVA; ^b^ chi-squared test. ^c^ two-sample *t* test.

**Table 2:** Differences in effective connectivity (EC) from Broca’s area to every whole-brain voxel among the three groups.

Brain Region	MNI Coordinates (X, Y, Z)	BA	Cluster Size (Voxel)	F Value	EC Z-Score (Mean ± SD)		
					Drug-Naïve	Medicated	HCs
Left HG	−39, −24, 9	48	47	9.75	0.26 ± 0.99 ^c^	0.13 ± 1.40 ^b^	−1.05 ± 0.77
Right putamen	30, 0, 6	-	39	10.58	−0.29 ± 0.61 ^a^	0.04 ± 0.83 ^c^	−0.86 ± 0.68
ACC	0, 36, 9	32	155	15.83	0.70 ± 1.47 ^c^	1.07 ± 1.18 ^c^	−1.32 ± 1.64

Abbreviations: MNI, Montreal Neurological Institute; BA, Brodmann area; SD, standard deviation; drug-naïve, unmedicated benign childhood epilepsy with centrotemporal spikes; medicated, medicated benign childhood epilepsy with centrotemporal spikes; HCs, healthy controls; HG, Heschl’s gyrus; ACC, anterior cingulate cortex. Superscripts indicate significance compared with HCs: ^a^ *p* < 0.05, ^b^ *p* < 0.01, ^c^ *p* < 0.001.

**Table 3:** Differences in effective connectivity (EC) from every whole-brain voxel to Broca’s area among the three groups.

Brain Region	MNI Coordinates (X, Y, Z)	BA	Cluster Size (Voxel)	F Value	EC Z-Score (Mean ± SD)		
					Drug-Naïve	Medicated	HCs
Left putamen	−18, 6, −6	-	33	8.20	−0.39 ± 2.12 ^b^	1.06 ± 1.55 ^d^	1.8 ± 2.02
Right putamen	24, −3, 3	-	48	8.93	0.86 ± 1.56 ^a^	0.34 ± 1.99 ^b^	2.47 ± 1.91
Left ACC	0, 36, 6	32	37	11.24	−0.55 ± 1.01 ^b^	−0.60 ± 0.84 ^c^	0.46 ± 0.79

Abbreviations: MNI, Montreal Neurological Institute; BA, Brodmann area; SD, standard deviation; drug-naïve, unmedicated benign childhood epilepsy with centrotemporal spikes; medicated, medicated benign childhood epilepsy with centrotemporal spikes; HCs, healthy controls; ACC, anterior cingulate cortex. Superscripts indicate significance compared with HCs: ^a^ *p* < 0.05, ^b^ *p* < 0.01, ^c^ *p* < 0.001. Significance compared with drug-naïve patients is indicated by ^d^ *p* < 0.05.

## Data Availability

The original contributions presented in the study are included in the article, and further inquiries can be directed to the corresponding authors.

## References

[1] Jiang S., Luo C., Huang Y., Li Z., Chen Y., Li X., Pei H., Wang P., Wang X., Yao D. (2020). Altered Static and Dynamic Spontaneous Neural Activity in Drug-Naïve and Drug-Receiving Benign Childhood Epilepsy with Centrotemporal Spikes. Front. Hum. Neurosci..

[2] Jiang L., Zhang T., Lv F., Li S., Liu H., Zhang Z., Luo T. (2018). Structural Covariance Network of Cortical Gyrification in Benign Childhood Epilepsy with Centrotemporal Spikes. Front. Neurol..

[3] Kessi M., Yan F., Pan L., Chen B., Olatoutou E., Li D., He F., Rugambwa T., Yang L., Peng J. (2021). Treatment for the Benign Childhood Epilepsy with Centrotemporal Spikes: A Monocentric Study. Front. Neurol..

[4] Danielsson J., Petermann F. (2009). Cognitive Deficits in Children with Benign Rolandic Epilepsy of Childhood or Rolandic Discharges: A Study of Children between 4 and 7 Years of Age with and without Seizures Compared with Healthy Controls. Epilepsy Behav..

[5] Li Y., Sun Y., Zhang T., Shi Q., Sun J., Xiang J., Chen Q., Hu Z., Wang X. (2020). The Relationship between Epilepsy and Cognitive Function in Benign Childhood Epilepsy with Centrotemporal Spikes. Brain Behav..

[6] McGinnity C.J., Smith A.B., Yaakub S.N., Gerbase S.W., Gammerman A., Tyson A.L., Bell T.K., Elmasri M., Barker G.J., Richardson M.P. (2017). Decreased Functional Connectivity within a Language Subnetwork in Benign Epilepsy with Centrotemporal Spikes. Epilepsia Open.

[7] Monjauze C., Tuller L., Hommet C., Barthez M.A., Khomsi A. (2005). Language in Benign Childhood Epilepsy with Centro-Temporal Spikes Abbreviated Form: Rolandic Epilepsy and Language. Brain Lang..

[8] Georgiopoulos C., Witt S.T., Haller S., Dizdar N., Zachrisson H., Engstrom M., Larsson E.M. (2019). A Study of Neural Activity and Functional Connectivity within the Olfactory Brain Network in Parkinson’s Disease. Neuroimage Clin..

[9] Jiang Y., Song L., Li X., Zhang Y., Chen Y., Jiang S., Hou C., Yao D., Wang X., Luo C. (2019). Dysfunctional White-Matter Networks in Medicated and Unmedicated Benign Epilepsy with Centrotemporal Spikes. Hum. Brain Mapp..

[10] Tu Y., Fu Z., Mao C., Falahpour M., Gollub R.L., Park J., Wilson G., Napadow V., Gerber J., Chan S.T. (2020). Distinct Thalamocortical Network Dynamics Are Associated with the Pathophysiology of Chronic Low Back Pain. Nat. Commun..

[11] Tracy J.I., Osipowicz K., Spechler P., Sharan A., Skidmore C., Doucet G., Sperling M.R. (2014). Functional Connectivity Evidence of Cortico-Cortico Inhibition in Temporal Lobe Epilepsy. Hum. Brain Mapp..

[12] Ma S., Calhoun V.D., Phlypo R., Adali T. (2014). Dynamic Changes of Spatial Functional Network Connectivity in Healthy Individuals and Schizophrenia Patients Using Independent Vector Analysis. Neuroimage.

[13] Koelewijn L., Hamandi K., Brindley L.M., Brookes M.J., Routley B.C., Muthukumaraswamy S.D., Williams N., Thomas M.A., Kirby A., Naude J.T.W. (2015). Resting-State Oscillatory Dynamics in Sensorimotor Cortex in Benign Epilepsy with Centro-Temporal Spikes and Typical Brain Development. Hum. Brain Mapp..

[14] Ofer I., Jacobs J., Jaiser N., Akin B., Hennig J., Schulze-Bonhage A., LeVan P. (2018). Cognitive and Behavioral Comorbidities in Rolandic Epilepsy and Their Relation with Default Mode Network’s Functional Connectivity and Organization. Epilepsy Behav..

[15] Xiao F., Li L., An D., Lei D., Tang Y., Yang T., Ren J., Chen S., Huang X., Gong Q. (2015). Altered Attention Networks in Benign Childhood Epilepsy with Centrotemporal Spikes (Bects): A Resting-State Fmri Study. Epilepsy Behav..

[16] Fang J., Chen S., Luo C., Gong Q., An D., Zhou D. (2017). Altered Language Network in Benign Childhood Epilepsy Patients with Spikes from Non-Dominant Side: A Resting-State Fmri Study. Epilepsy Res..

[17] Kim H.J., Lee J.H., Park C.H., Hong H.S., Choi Y.S., Yoo J.H., Lee H.W. (2018). Role of Language-Related Functional Connectivity in Patients with Benign Childhood Epilepsy with Centrotemporal Spikes. J. Clin. Neurol..

[18] Besseling R.M., Jansen J.F., Overvliet G.M., van der Kruijs S.J., Vles J.S., Ebus S.C., Hofman P.A., Louw A., Aldenkamp A.P., Backes W.H. (2013). Reduced Functional Integration of the Sensorimotor and Language Network in Rolandic Epilepsy. Neuroimage Clin..

[19] Besseling R.M., Overvliet G.M., Jansen J.F., van der Kruijs S.J., Vles J.S., Ebus S.C., Hofman P.A., de Louw A.J., Aldenkamp A.P., Backes W.H. (2013). Aberrant Functional Connectivity between Motor and Language Networks in Rolandic Epilepsy. Epilepsy Res..

[20] Oser N., Hubacher M., Specht K., Datta A.N., Weber P., Penner I.K. (2014). Default Mode Network Alterations During Language Task Performance in Children with Benign Epilepsy with Centrotemporal Spikes (Bects). Epilepsy Behav..

[21] Park C.H., Choi Y.S., Kim H.J., Chung H.K., Jung A.R., Yoo J.H., Lee H.W. (2018). Interactive Effects of Seizure Frequency and Lateralization on Intratemporal Effective Connectivity in Temporal Lobe Epilepsy. Epilepsia.

[22] Ewen J.B., Lakshmanan B.M., Hallett M., Mostofsky S.H., Crone N.E., Korzeniewska A. (2015). Dynamics of Functional and Effective Connectivity within Human Cortical Motor Control Networks. Clin. Neurophysiol..

[23] Fan X., Yan H., Shan Y., Shang K., Wang X., Wang P., Shan Y., Lu J., Zhao G. (2016). Distinctive Structural and Effective Connectivity Changes of Semantic Cognition Network across Left and Right Mesial Temporal Lobe Epilepsy Patients. Neural. Plast.

[24] Jiang L., Ma X., Liu H., Wang J., Zhang J., Zhang G., Li S., Zhang T. (2021). Aberrant Dynamics of Regional Coherence Measured by Resting-State Fmri in Children with Benign Epilepsy with Centrotemporal Spikes (Bects). Front. Neurol..

[25] Horwitz B., Amunts K., Bhattacharyya R., Patkin D., Jeffries K., Zilles K., Braun A.R. (2003). Activation of Broca’s Area During the Production of Spoken and Signed Language: A Combined Cytoarchitectonic Mapping and Pet Analysis. Neuropsychologia.

[26] Proposal for Revised Classification of Epilepsies and Epileptic Syndromes (1989). Commission on Classification and Terminology of the International League against Epilepsy. Epilepsia.

[27] Scheffer I.E., Berkovic S., Capovilla G., Connolly M.B., French J., Guilhoto L., Hirsch E., Jain S., Mathern G.W., Moshe S.L. (2017). Ilae Classification of the Epilepsies: Position Paper of the Ilae Commission for Classification and Terminology. Epilepsia.

[28] Lin F.H., Hara K., Solo V., Vangel M., Belliveau J.W., Stufflebeam S.M., Hamalainen M.S. (2009). Dynamic Granger-Geweke Causality Modeling with Application to Interictal Spike Propagation. Hum. Brain Mapp..

[29] Chen S., Fang J., An D., Xiao F., Chen D., Chen T., Zhou D., Liu L. (2018). The Focal Alteration and Causal Connectivity in Children with New-Onset Benign Epilepsy with Centrotemporal Spikes. Sci. Rep..

[30] Bassett D.S., Wymbs N.F., Rombach M.P., Porter M.A., Mucha P.J., Grafton S.T. (2013). Task-Based Core-Periphery Organization of Human Brain Dynamics. PLoS Comput Biol.

[31] Fedorenko E. (2014). The Role of Domain-General Cognitive Control in Language Comprehension. Front. Psychol..

[32] Wallace M.P., Lee K. (2020). Examining Second Language Listening, Vocabulary, and Executive Functioning. Front. Psychol..

[33] Fu C., Aisikaer A., Chen Z., Yu Q., Yin J., Yang W. (2021). Different Functional Network Connectivity Patterns in Epilepsy: A Rest-State Fmri Study on Mesial Temporal Lobe Epilepsy and Benign Epilepsy with Centrotemporal Spike. Front. Neurol..

[34] Fratantoni J.M., DeLaRosa B.L., Didehbani N., Hart J., Kraut M.A. (2017). Electrophysiological Correlates of Word Retrieval in Traumatic Brain Injury. J. Neurotrauma.

[35] Choi H.S., Chung Y.G., Choi S.A., Ahn S., Kim H., Yoon S., Hwang H., Kim K.J. (2019). Electroencephalographic Resting-State Functional Connectivity of Benign Epilepsy with Centrotemporal Spikes. J. Clin. Neurol..

[36] Liao W., Zhang Z., Pan Z., Mantini D., Ding J., Duan X., Luo C., Wang Z., Tan Q., Lu G. (2011). Default Mode Network Abnormalities in Mesial Temporal Lobe Epilepsy: A Study Combining Fmri and Dti. Hum. Brain Mapp..

[37] Ciumas C., Montavont A., Ilski F., Laurent A., Saignavongs M., Lachaux J.P., de Bellescize J., Panagiotakaki E., Ostrowsky-Coste K., Herbillon V. (2020). Neural Correlates of Verbal Working Memory in Children with Epilepsy with Centro-Temporal Spikes. Neuroimage Clin..

[38] Cadena E.J., White D.M., Kraguljac N.V., Reid M.A., Maximo J.O., Nelson E.A., Gawronski B.A., Lahti A.C. (2018). A Longitudinal Multimodal Neuroimaging Study to Examine Relationships between Resting State Glutamate and Task Related Bold Response in Schizophrenia. Front. Psychiatry.

[39] Burhan A.M., Marlatt N.M., Palaniyappan L., Anazodo U.C., Prato F.S. (2015). Role of Hybrid Brain Imaging in Neuropsychiatric Disorders. Diagnostics.

[40] Thothathiri M., Rattinger M., Trivedi B. (2017). Cognitive Control During Sentence Generation. Cogn. Neurosci..

[41] Weber K., Micheli C., Ruigendijk E., Rieger J.W. (2019). Sentence Processing Is Modulated by the Current Linguistic Environment and a Priori Information: An Fmri Study. Brain Behav..

[42] Luo C., Zhang Y., Cao W., Huang Y., Yang F., Wang J., Tu S., Wang X., Yao D. (2015). Altered Structural and Functional Feature of Striato-Cortical Circuit in Benign Epilepsy with Centrotemporal Spikes. Int. J. Neural. Syst..

[43] Grillner S., Hellgren J., Menard A., Saitoh K., Wikstrom M.A. (2005). Mechanisms for Selection of Basic Motor Programs--Roles for the Striatum and Pallidum. Trends Neurosci..

[44] Badawy R.A., Lai A., Vogrin S.J., Cook M.J. (2013). Subcortical Epilepsy?. Neurology.

